# Synthesis and Electro-Magneto-Mechanical Properties of Graphene Aerogels Functionalized with Co-Fe-P Amorphous Alloys

**DOI:** 10.3390/mi7070117

**Published:** 2016-07-12

**Authors:** Guang-Ping Zheng, Xi Lu, Zhuo Han

**Affiliations:** Department of Mechanical Engineering, Hong Kong Polytechnic University, Hung Hom, Kowloon, Hong Kong, China; xi.lu@connect.polyu.hk (X.L.); zhuo.han@polyu.edu.hk (Z.H.)

**Keywords:** graphene aerogel, electro-magneto-mechanical properties, sensors, amorphous alloys

## Abstract

Graphene aerogels (GAs) are functionalized with Fe-Co-P alloy using an electro-deposition method. The Fe-Co-P alloy coated on the graphene nanosheets is found to possess an amorphous structure and a nanoporous architecture of GAs. The electro-mechanical properties of GAs are significantly affected by the Fe-Co-P nanoparticles embedded inside GAs. The electro-mechanical responses of GA/Fe-Co-P nanoporous hybrid structures are sensitive to an applied magnetic field, demonstrating that they are promising for electro-magneto-mechanical applications. The light-weight, high-strength and nanoporous GAs functionalized with Fe-Co-P amorphous alloys are desirable sensors, actuators, and nano-electro-mechanical systems that could be controlled or manipulated by mechanical, electric and magnetic fields.

## 1. Introduction

Although graphene is a two-dimensional (2D) material with extraordinary properties, an immediate challenge in the applications of graphene is to develop bulk graphene architectures consisting of graphene nanosheets. Recently, three-dimensional (3D) graphene nanoporous structure, especially the graphene aerogel (GA), which is an aggregation network of 2D graphene, has attracted much attention [1,2,3,4,5,6,7,8]. Since GAs exhibit super-elasticity, high electro-mechanical sensitivity, ultra-light weight (density < 1.0 mg/cm^3^) and ultra-large surface area (>200 m^2^/g), they are very promising to be used as devices and structures in micro-machines and nano-devices, such as sensors, actuators, and devices for energy storage and water purification [9,10,11,12,13,14,15,16,17,18].

Because of their exceptionally large surface areas (as large as 2630 m^2^/g) and good electrical conductivity (~10^2^ S/m), GAs are desirable supports for micro- and nano-sized metals, oxides, magnets, photo-catalysts and electro-catalysts, which have significantly enhanced performances compared with their pristine counterparts [9,10,11,12,13,14,15,16,17,18]. In particular, owing to their super-elasticity (up to 200% in compression) and stress-dependent electrical conductivity, GAs are suitable nano-electro-mechanical sensors and devices for various applications, such as micro- or nano-sized strain gauges, stress and pressure sensors. However, there are two major drawbacks of GAs which will prevent them from wide-spread applications. First, the high porosity of GAs usually results in significant reduction of their mechanical strength [1,2,3,4]. Second, the graphene nanosheets in GAs are reduced graphene oxides, which have low electrical conductivity [5,6,7]. Hence, graphene aerogel with improved mechanical and electrical performances are urgently needed.

In this work, we investigate GAs functionalized with Fe-Co-P alloys. The nanoporous structures of samples and the phases of Fe-Co-P embedded inside GAs are characterized. The mechanical and magnetic properties of the samples are measured. The relations between the electrical resistances of samples and the applied stresses and magnetic fields are determined, and the electro-magneto-mechanical applications of GA/Co-Fe-P hybrid structures are discussed.

## 2. Experimental Methods

Graphene oxides (GOs) were prepared by a modified Hummers method from natural graphite flakes. Graphene hydrogels (GHs) were fabricated via a hydrothermal process using GOs as the starting materials [2,3]. Firstly, 2 mg·mL^−1^ GO solution was hydrothermally treated in a Teflon-lined stainless-steel autoclave at 180 °C for 20 h to obtain the GH. Ammonia solution was then added to the GHs, which were subsequently treated in another autoclave at 90 °C for 1 h. After the hydrothermal treatment, the GHs were put in a freeze drying machine and were cooled to −70 °C. Supercritical drying of GHs occurred under a pressure of 10 kPa for 2–3 days. Finally cylindrical graphene aerogels with a height of ~15 mm were obtained. Those 3D graphene monoliths have a diameter of about 6–12 mm.

The GA/Co-Fe-P hybrid structures were synthesized by electroplating Co-Fe-P alloys into the GAs. The FeSO_4_·7H_2_O (4.171 g), CoSO_4_·7H_2_O (2.812 g), C_6_H_5_Na_3_O_7_·2H_2_O (14.705 g), H_3_BO_3_ (7.729 g), NaH_2_PO_2_·H_2_O (5.3 g) were dissolved into 250 mL deionized water to form an electrolyte. The PH value of the electrolyte was adjusted to 5–6 using H_2_SO_4_. The temperature of the electrolyte was maintained at 60 °C. A GA with a height of about 5–10 mm was mounted on a titanium plate, which was used as a working electrode operating at a voltage of 1.0 V with respect to the reference electrode of saturated calomel electrode (SCE). The samples were electroplated for 2, 4, 8, and 14 h, which were denoted as Sample-h2, h4, h8 and h14, respectively. After the electro-deposition the GAs were peeled off the titanium plate. The as-prepared GA/Co-Fe-P hybrid structures were then immersed into deionized water for two days to remove the electrolyte remained inside the GA. The samples were then dried by freeze drying at conditions of −50 °C and 20 Pa for 72 h.

XRD patterns of the samples were taken by an X-ray diffractometer (Philips PW3040/60, Philips, Amsterdam, The Netherlands) with nickel filtered Cu Kα radiation (λ = 0.154 nm). Scanning Electron Microscopy (SEM, JEOL JSM-6490, JEOL Inc., Peabody, MA, USA) operated at 20 kV was utilized to observe the microstructures of the samples. The SEM was equipped with energy dispersive X-ray (EDX, Peabody, MA, USA) analysis which was used to determine the samples’ compositions. A surface area and porosity analyzer (Micromeritics ASAP 2420, Micromeritics Instrument Corporation, Norcross, GA, USA) was used to analyze the porous nature of the samples. The nitrogen adsorption-desorption isotherms were obtained at −196 °C and the surface areas were calculated using the Barrett−Joyner−Halenda (BJH) method. Raman experiments were carried out on a Raman Station 400/400F with a resolution of 1.0 cm^−1^.

A mechanical testing machine (Materials Testing, ZWICK Z2.5^TH^, Zwick Techonology & Instrument Co. Ltd., Shanghai, China) was used to measure the compressive strength of samples, which had been polished into cylindrical shape with a height of 5 mm and a diameter of 3–4 mm. The compressive strain rate was 0.01 min^−1^. The electrodes of the samples were prepared by coating silver paste at their top and bottom surfaces. The currents of the sample under the applied voltages of 0–2 V were measured by the ferroelectric test system (TF2000E, aixACCT, Aachen, Germany) under the leakage current measuring mode. The electrical resistances of samples under compression were determined by the Ohm’s law. The magnetic properties of the samples were characterized by the magnetic hysteresis loops, which were measured by a vibrating sample magnetometer (VSM, Lakeshore Model 7300, Lake Shore Cryotronics Inc., Westerville, OH, USA). The same procedures were applied to measure the electro-mechanical responses of the samples under an applied magnetic field which was supplied by a solenoid magnet.

## 3. Results and Discussion

### 3.1. Characterizations of Samples

**Figure 1:**
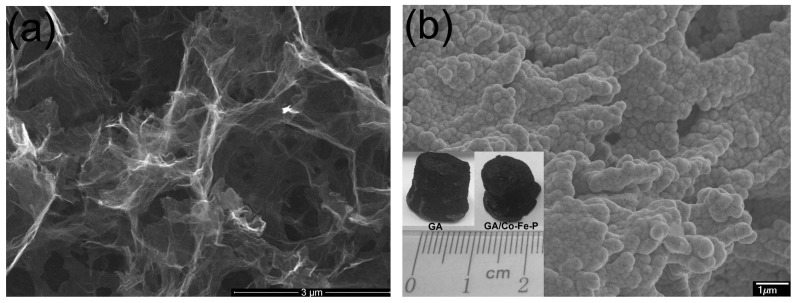
SEM images of GA (**a**) and GA/Co-Fe-P (Sample-h14) (**b**). The inset in (**b**) shows the typical size and shape of GA and GA/Co-Fe-P samples.

**Figure 2:**
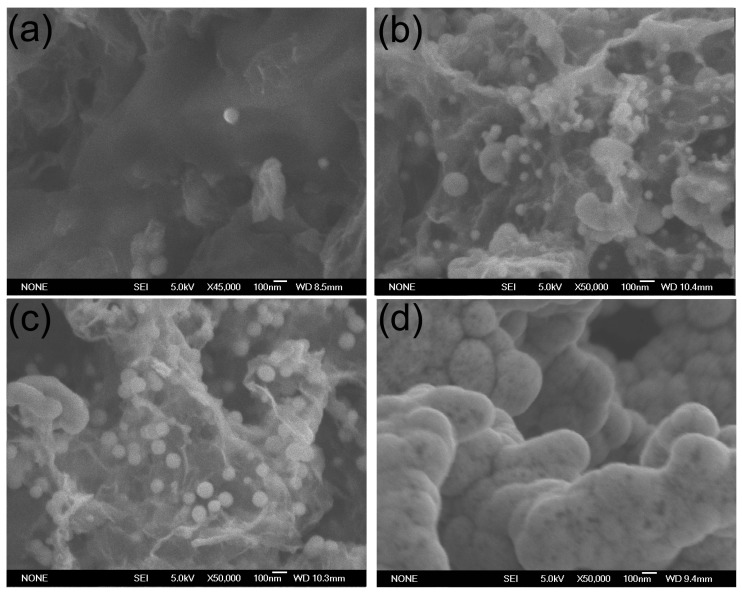
SEM images of GA/Co-Fe-P samples: (**a**) Sample-h2; (**b**) Sample-h4; (**c**) Sample-h8; (**d**) Sample-h14.

SEM images of typical GAs and GA/Co-Fe-P samples are shown in Figure 1a,b. The shape of GA/Co-Fe-P sample is not much different with the cylindrical shape of monolith GA sample, as shown in the inset in Figure 1b. It can be seen in Figure 1 that the nanoporous architectures of GA are preserved in the GA/Co-Fe-P hybrid structures (Sample-h14) and the Co-Fe-P fully coated on the graphene nanosheets consists of a lot of nanoparticles. Figure 2 shows the SEM images of all GA/Co-Fe-P samples. The sizes and contents of Co-Fe-P nanoparticles in the hybrid structures can be found to increase with increasing deposition time. It seems that the Co-Fe-P alloys fully coated on the graphene nanosheets in Sample-h14 could result from the growth of Co-Fe-P nanoparticles whose sizes can be as small as 20–30 nm, as shown in Figure 2a for Sample-h2.

**Figure 3:**
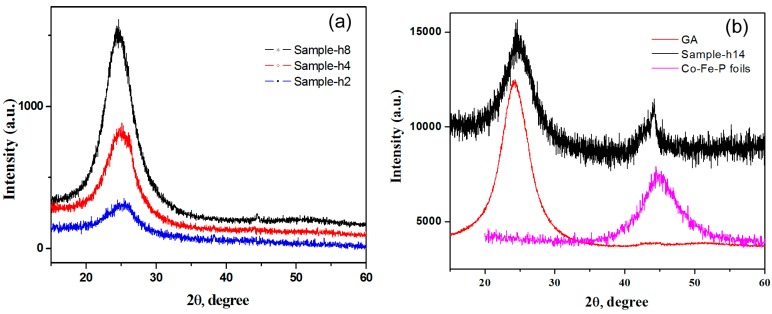
XRD patterns of GA and GA/Co-Fe-P samples: (**a**) Sample-h2, Sample-h4 and Sample-h8; (**b**) GA, Co-Fe-P foils and Sample-h14.

Figure 3 shows the XRD patterns of GA/Co-Fe-P samples. In comparison with those of GA and Co-Fe-P foils prepared by the same electrodeposition conditions, the XRD peaks for Sample-h14 demonstrate that the Co-Fe-P alloys coated on the graphene nanosheets is amorphous, as shown in Figure 3b. The intensity of the broad peak at 44.7° for the amorphous phase of Co-Fe-P indicates that its content dramatically increases when the electrodeposition time is larger than 8 h, as shown in Figure 3a for Sample-h2, h4 and h8 and in Figure 3b for Sample-h14.

Table 1 lists the results of analysis on the nanoporous structures of GA/CO-Fe-P samples. As indicated by the specific surface area (SSA) and volume (V_total_), the Co-Fe-P nanoparticles embedded on the graphene nanosheets significantly reduce the SSA and V_total_ of Sample-h2 and Sample-h4. With the evolution of Co-Fe-P nanoparticles throughout the graphene nanosheets, the SSA and V_total_ of Sample-h8 and Sample-h14 increase, suggesting that the Co-Fe-P amorphous alloy tends to form film-like coating on the surfaces of graphene nanosheets when the content of Co-Fe-P increases. As listed in Table 1, the average size of nano-pores of the hybrid structures increases with increasing content of Co-Fe-P.

**Figure 4:**
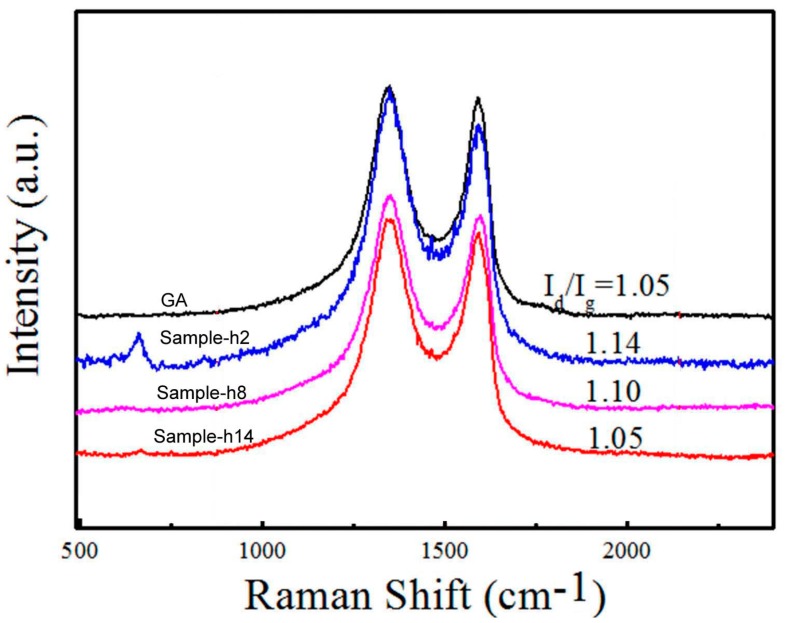
Raman spectra of GA and GA/Co-Fe-P samples. I_d_/I_g_ is the ratio of intensities of D- and G-bands.

Raman spectra of the GA/Co-Fe-P samples are shown in Figure 4. The D-band (1327 cm^−1^) represents the defects in the graphene or amorphous carbon; the G-band (1593 cm^−1^) is caused by the in-plane vibration of graphite with an E_2g_-symmetry intra-layer mode. The intensity ratio of D-band to G-bands (I_d_/I_g_) is higher in Sample-h2 than those of any other samples, suggesting that the defective carbon structures can be induced in Sample-h2 where Co-Fe-P nanoparticles with sizes of 20–30 nm might interact with the graphene nanosheets or their junctions. Becasue the surfaces of Co-Fe-P nanoparticles are less active toward the bonding with carbon atoms of the graphene nanosheets when the sizes of nanoparticles are increased, therefore the ratio I_d_/I_g_ could decrease to that of graphene with increasing sizes of nanoparticles.

### 3.2. The Electro-Mechanical Properties of GA/Co-Fe-P Samples

The compressive strength of Sample-h2, h4, h8 and h14 are determined from the compression tests to be 0.2, 3.8, 22 and 620 kPa, respectively. Except for Sample-h2 whose fracture strength is smaller than that of GA (0.6 kPa), the GA/Co-Fe-P samples exhibit much improved strength as compared with the monolith GA. The small mechanical strength of Sample-h2 might be related with the disorder graphene nanosheets induced by the embedded Co-Fe-P nanoparticles with sizes of 20–30 nm. When the Co-Fe-P amorphous alloys fully coat the graphene nanosheets inside GA, as shown in Figure 1b for Sample-h14, the mechanical properties of GA/Co-Fe-P hybrid structure are mainly determined by the Co-Fe-P amorphous alloys. Therefore the fracture strength of GA/Co-Fe-P hybrid structure increases with the increasing content of Co-Fe-P in a non-linear manner.

**Figure 5:**
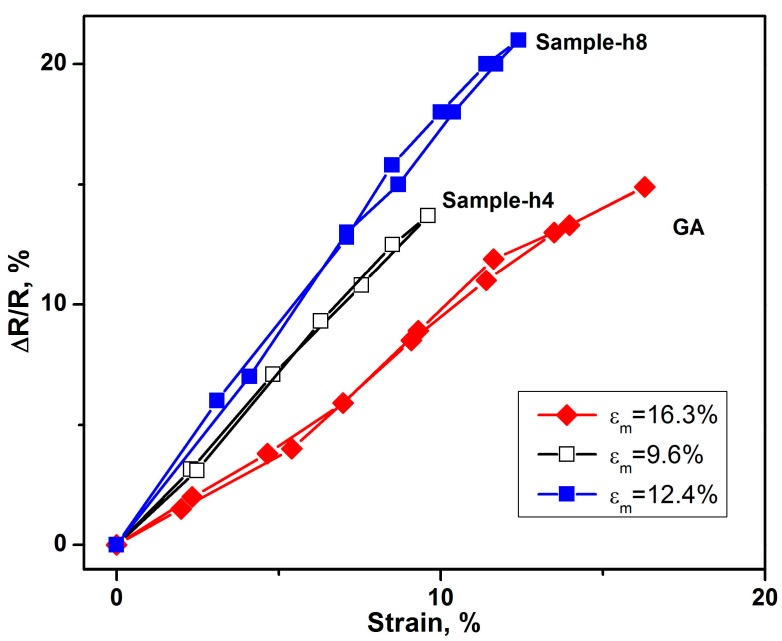
The relative changes of resistances of GA and GA/Co-Fe-P samples under compressive strains in a loading (from 0 to ε_m_) and un-loading (from ε_m_ to 0) cycle. ε_m_ denotes the maximum applied strain.

The GA/Co-Fe-P hybrid structures are placed along the horizontal direction between two copper plates. Thin copper wires are attached to the two copper plates which are separately contacted with the top and bottom electrodes of the samples. The copper plates are driven to move horizontally in the opposite directions, leading to the compression on the samples. When the samples are compressed by a uniaxial compressive strain ε, the electrical resistances R(ε) of the samples are measured. Figure 5 shows the repeatable change of electrical resistance ∆R = |R − R(ε)| of the GA/Co-Fe-P samples (Sample-h4 and Sample-h8) with ε, which is cyclic between 0 and ε_m_. R is the electrical resistance at ε = 0. A linear relation between ∆R/R and ε changing from 0 to ε_m_ and then back to 0 can be observed, the ∆R/R~ε relations for Sample-h2 and Sample-h14 are not shown either because they have ε_m_ < 2% (Sample-h2) or the change in ∆R/R is less than 1% (Sample-h14).

The repeatable changes of electrical resistances under applied strains in Sample-h4 and Sample-h8 indicate that they have better electro-mechanical performances or sensitivities compared to monolith GA. Combined with its high mechanical strength and light weight, Sample-h8 has the best performance in nano-electro-mechanical applications.

### 3.3. The Electro-Magneto-Mechanical Properties of GA/Co-Fe-P Samples

**Figure 6:**
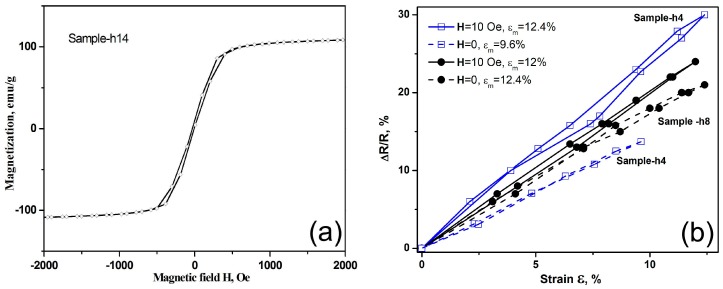
(**a**) Magnetic hysteresis loop of the GA/Co-Fe-P sample; (**b**) The relative changes of resistances of GA/Co-Fe-P samples under compressive strains in a loading (from 0 to ε_m_) and un-loading (after ε_m_ to 0) cycle, with and without an applied magnetic field H. ε_m_ denotes the maximum applied strain.

The electro-deposited Co-Fe-P alloy foils have been well studied to have excellent soft magnetic properties [19]. Although in the GA/Co-Fe-P hybrid structures only Sample-h14 is found to exhibit magnetic properties which are detectable by the VSM, as shown in Figure 6a, other GA/Co-Fe-P hybrid structures containing Fe-Co-P magnetic materials could be also sensitive to an applied magnetic field. Therefore, the repeatable changes of electrical resistances of the GA/Co-Fe-P samples under applied strains could be affected by the applied magnetic fields.

Figure 6b shows the ∆R/R~ε relations for Sample-h4 and Sample-h8 with and without a magnetic field of 10 Oe applied along the compression direction. Both samples have enhanced electro-mechanical sensitivity when a magnetic field is applied. Remarkably, the electro-mechanical responses under a magnetic field can be as high as 3 times of those of Sample-h4 without an applied magnetic field. Although the effects of nanoporous structures of GA/Co-Fe-P and the sizes of Co-Fe-P nanoparticles embedded on the graphene nanosheets on the electro-magneto-mechanical properties are yet to be further explored, the results suggest that the GA/Co-Fe-P hybrid structures have outstanding electro-magneto-mechanical responses and could be promising nano-electro-mechanical systems controlled or manipulated by multiple applied fields.

## 4. Conclusions

We investigate GAs functionalized with Fe-Co-P alloys, which are promising materials for nano-electro-mechanical systems and nano-devices. The GA/Co-Fe-P hybrid structures synthesized by electrodeposition are found to have nanoporous structures and contain amorphous Fe-Co-P nanoparticles embedded on the graphene nanosheets. The relations between the electrical resistances of samples and the applied strains and magnetic fields are determined. The results demonstrate that GAs functionalized with Fe-Co-P amorphous alloys are suitable for electro-magneto-mechanical applications in micro-machine and nano-devices.

## Figures and Tables

**Table 1:** The composition, specific surface area (SSA) and volume (V_total_), and pore width of GA and GA/Co-Fe-P samples.

Samples	Co/Fe/P Mole Fraction	SSA (m^2^·g^−1^)	V_total_ (cm^3^/g)	Pore Width (nm)
GA	-	370.9022	0.449374	5.81898
Sample-h2	-	70.225	0.071297	4.06106
Sample-h4	-	10.7219	0.020687	7.71771
Sample-h8	32.5/32.7/33.8	44.9051	0.11327	10.08971
Sample-h14	31.2/25.7/43.1	55.5464	0.154443	11.12175
